# User Interest in Digital Health Technologies to Encourage Physical Activity: Results of a Survey in Students and Staff of a German University

**DOI:** 10.2196/mhealth.7192

**Published:** 2017-04-19

**Authors:** Annett Salzwedel, Sophie Rabe, Thomas Zahn, Julia Neuwirth, Sarah Eichler, Kathrin Haubold, Anne Wachholz, Rona Reibis, Heinz Völler

**Affiliations:** ^1^ Center of Rehabilitation Research University of Potsdam Potsdam Germany; ^2^ GeWINO - Health Research Institute AOK Nordost - Die Gesundheitskasse Berlin Germany; ^3^ Cardiological Outpatient Clinic Am Park Sanssouci Potsdam Potsdam Germany

**Keywords:** physical activity, telemedicine, primary prevention, healthy lifestyle

## Abstract

**Background:**

Although the benefits for health of physical activity (PA) are well documented, the majority of the population is unable to implement present recommendations into daily routine. Mobile health (mHealth) apps could help increase the level of PA. However, this is contingent on the interest of potential users.

**Objective:**

The aim of this study was the explorative, nuanced determination of the interest in mHealth apps with respect to PA among students and staff of a university.

**Methods:**

We conducted a Web-based survey from June to July 2015 in which students and employees from the University of Potsdam were asked about their activity level, interest in mHealth fitness apps, chronic diseases, and sociodemographic parameters.

**Results:**

A total of 1217 students (67.30%, 819/1217; female; 26.0 years [SD 4.9]) and 485 employees (67.5%, 327/485; female; 42.7 years [SD 11.7]) participated in the survey. The recommendation for PA (3 times per week) was not met by 70.1% (340/485) of employees and 52.67% (641/1217) of students. Within these groups, 53.2% (341/641 students) and 44.2% (150/340 employees)—independent of age, sex, body mass index (BMI), and level of education or professional qualification—indicated an interest in mHealth fitness apps.

**Conclusions:**

Even in a younger, highly educated population, the majority of respondents reported an insufficient level of PA. About half of them indicated their interest in training support. This suggests that the use of personalized mobile fitness apps may become increasingly significant for a positive change of lifestyle.

## Introduction

There is considerable evidence that physical activity (PA) has a positive effect on health [1,2]. A sedentary lifestyle, on the other hand, increases the risk of developing cardiovascular, metabolic, or malignant diseases and leads to a reduction in life expectancy [3,4]. According to the World Health Organization (WHO), 150 min per week of moderately intensive endurance activity is currently recommended as the minimum level for promoting health in adults, both in primary and in secondary and tertiary prevention [5]. Although many individuals are already well aware of the positive effects of PA, a large part of the population does not succeed in continuously implementing the recommendations for increasing PA in their everyday lives [5-7].

The increasingly available mobile health (mHealth) trackers such as wearables or mobile phone apps are usually developed with the motivational aspect in mind and offered to a wide range of potential users. They are an opportunity for improving self-activation and can help overcome implicit barriers for engaging in PA. Over 100,000 of these apps are already available [8]. Many of these apps aim to increase physical capacity and include features such as heart rate monitor, pedometer, activity instructions, and activity monitor [9]. In addition, they often implement behavioral components such as self-check, feedback mechanisms, or social support features, which can help optimize starting and continuing PA [10]. Depending on their content, mHealth apps can support the intention to become physically active or to increase the PA level. Moreover, the transfer into everyday life as well as the long-term maintenance of PA may be ensured. Those phases meet the stages of the transtheoretical model (preparation, action, maintenance) [11]. Concerning the development of model-based, effective apps, the needs of potential users must be considered. However, the basic condition in such apps remains the interest of the target group.

Meta-analyses and systematic reviews that have been conducted were able to prove the effect of mHealth monitors with respect to increasing PA, but according to the authors, long-term randomized controlled trials, especially of larger samples, are lacking thus far [12-14]. Furthermore, the training recommendations that are implemented in most of the apps developed until now are not sufficiently evidence-based [9,10,13,15,16]. Additionally, the majority of mHealth systems are not medical products that are clinically evaluated. Therefore, validity and reliability of these systems cannot be assumed [17-20].

But, due to the potential benefit of mHealth monitors for increasing PA, they are particularly significant for stakeholders in the health care system, especially insurance companies. However, before noncommercial apps are developed by health insurers, the acceptance and general interest of potential users and the special interest in individual features of the systems (eg, providing information, documenting measured values, reminders, and instructions) should be determined.

The aim of this study was the explorative, nuanced determination of the interest in mHealth apps with respect to PA among students and staff of a university.

## Methods

### Survey Design and Implementation

In the period from June to July 2015, an Web-based survey was conducted among students and staff of the University of Potsdam. The survey mailing list referred to the students and employee database of the University of Potsdam. Mail addresses of all students, scientific and administration employees were included. The cover letter of the survey mail informed potential participants about the content and aim of the survey, their voluntary participation, the required time, and contained a hyperlink that guided participants to the survey. The survey mail was sent once from the administration department of the University of Potsdam. The survey instrument was created in “UP survey,” which is a platform of the University of Potsdam offering a toolset based on the software Solutions QUAMP (QUAMP qEducation Software, Sociolutions GmbH Potsdam).

The standardized questionnaire included 35 questions (90 items) about PA, the use of or interest in digital fitness apps, sociodemographic parameters such as age, gender, body mass index (BMI), and level of education, and presence of a chronic disease. PA was assessed by questioning patients about their PA during the last 3 months. The question was specified with the terms PA, sports, or fitness during leisure time that led at least to a low increase of respiratory and heart rate. Nordic walking, ball games, jogging, bicycling, swimming, aerobic, and badminton were cited as examples. All questions about PA in the survey were related to this definition.

Chronic disease was assessed by asking participants if they suffer from a chronic disease or a longer lasting health concern persisting for more than six months.

The arrangement of the questions was the result of an optimization process that took the perspective of the surveyed persons into account along with validity aspects and technical requirements (filter questions). By using filter questions, individual questions could be varied depending on the situation and—for certain subgroups of participants—redundant topics were avoided. Groups that were more and less interested in PA or digital media were thus addressed specifically. Accordingly, the questionnaires included sections of questions for participants who were more and less interested in PA and who were additionally differentiated according to the use of or interest in digital fitness apps.

The study used established terminology that was adapted to the target groups in the study or to the topics of this study. Questions about PA and sociodemographic data were based largely on the health survey of the Robert Koch Institute [6]. Questions about digital activity trackers and health or fitness apps were based on a study of mobile software apps [15].

### Survey Functionality and Analysis

The programmed survey was tested several times to ensure its functionality and usability. Using IP-Check, duplicates could be excluded in the analysis. No personal data were collected. Data were anonymously transmitted and descriptively analyzed. The results of the descriptive statistics were presented as percentages for categorical variables and as mean values (standard deviation) for metric variables. Group differences regarding the level of PA (active: ≥3 days/week, less active: 1-2 days/week, inactive: <1 day/week) and the interest in mHealth apps were determined using the chi-square test for categorical variables, the Mann-Whitney *U* test for ordinal variables, and the *t* test for metric variables. The level of significance was determined as *P*<.05. The statistical analyses were calculated using SPSS (IBM SPSS Statistics for Windows, version 22.0; IBM Corporation).

## Results

### Study Participants

A total of 18,961 students and 2621 employees of the University of Potsdam were invited to take part in the Web-based survey. After data processing and correction, a total of 1217 data sets for students (6.42%) and 485 data sets for staff (18.50%) were evaluated.

Around one-fifth of the students and one-third of employees reported suffering from a chronic disease; those affected within each group were significantly older than healthy members of the group (students: 26.8 vs 25.7 years, *P*=.01; staff: 46.1 vs 41.0 years, *P*=.001). In addition, 26.05% (317/1217) of participating students and over 43.1% (209/485) of staff were overweight to obese (BMI ≥25 kg/m^2^; Table 1).

**Table 1:** Basic characteristics of study participants.

Characteristics		Students (n=1217)	Staff (n=485)
Age in years, mean (SD)		26.0 (4.9)	42.7 (11.7)
Gender (male, n %)		398 (32.70)	158 (32.5)
High school graduate, n (%)		N/A^a^	416 (85.8)
University degree, n (%)		N/A^a^	357 (73.6)
Chronic disease^b^, n (%)		251 (20.62)	145 (29.9)
**BMI^c^** **(kg/m^2^** **), mean (SD)**		22.7 (9.5)	24.1 (3.6)
	Normal weight, n (%)	900 (74.00)	276 (56.9)
	Overweight, n (%)	262 (21.53)	165 (34.0)
	Obese, n (%)	55 (4.52)	44 (9.1)
**Extent of PA^d^** **, n (%)**			
	≥1 day/week	1095 (90.00)	397 (81.9)
	≥3 days/week	576 (47.33)	145 (29.9)

^a^N/A: not applicable.

^b^Chronic diseases or long-term health problems lasting at least six months.

^c^Body mass index (BMI) classes: normal weight <25 kg/m², overweight 25 to <30 kg/m², obese ≥30 kg/m².

^d^PA: physical activity.

**Figure 1:**
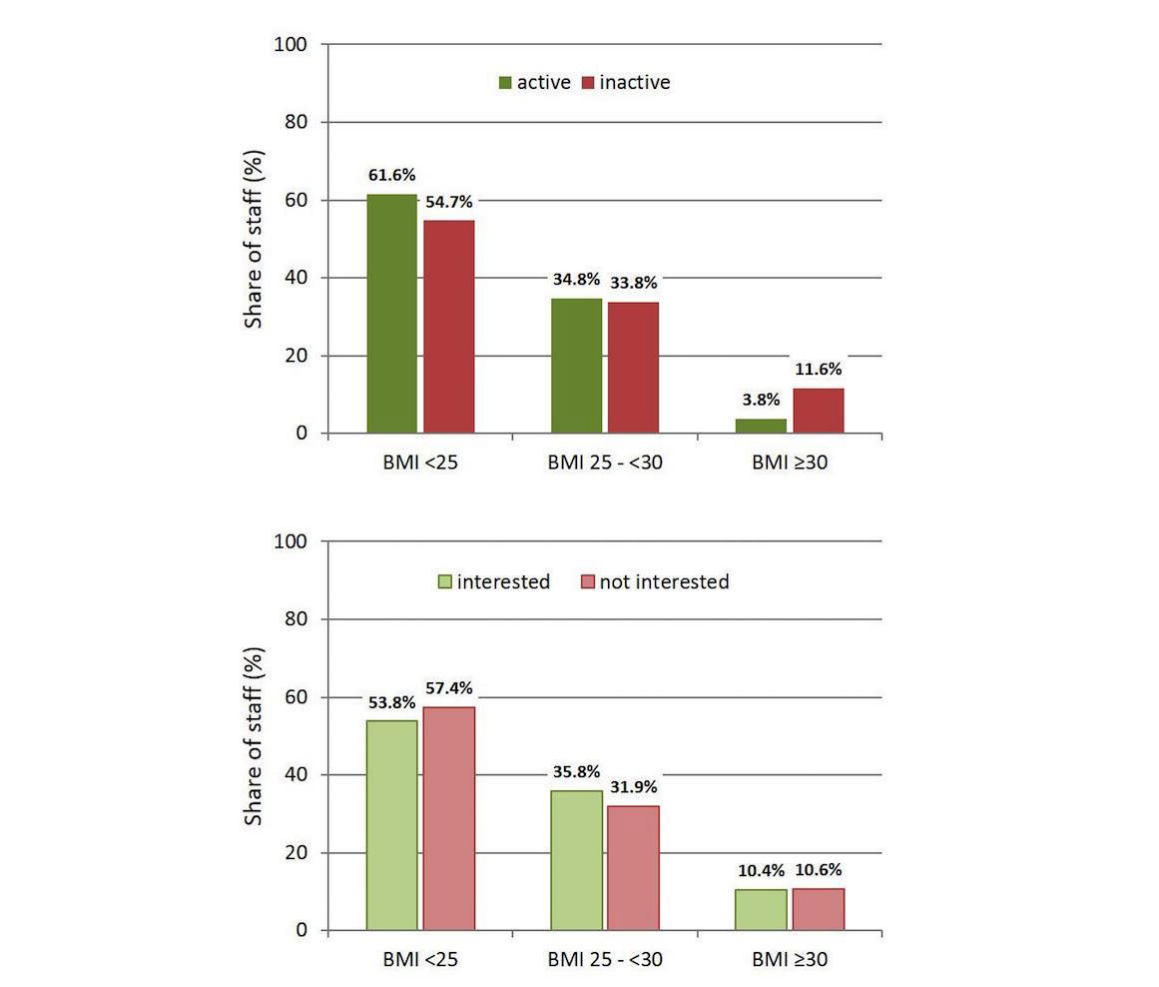
Physical activity and interest in mobile fitness apps with regard to the body mass index (BMI) class of the university staff surveyed. (Top) BMI and physical activity (≥3 days/week). (Bottom) BMI and the interest in mobile fitness apps to increase physical activity in less active individuals (<3 days/week). BMI is usually expressed in kilogram per square meter (kg/m²).

Some 90.00% (1095/1217) of students and 82% (398/485) of staff engaged in PA at least one day a week; nearly half of the students and one-third of the staff met the WHO’s recommendations for PA (≥3 days or 150 min per week). The level of PA was dependent on gender, that is, among the physically active (eg, ≥3 days/week) participants, the percentage of males was higher both for students and for staff than in the respective group of less active (eg, 1-2 days/week) or not active (eg, <1 day/week) participants (students: 38.8 [224/576] vs 27.2% [174/641], *P*<.001; staff: 39.3 [57/145] vs 29.5% [100/340], *P*=.04). Overall, 56.5% (225/398) of the male and 43.3% (355/819) of the female students reported that they engaged in PA at least 3 times a week. Among staff members, 36.7% (58/158) of the men and 27.2% (89/327) of the women met these recommendations for PA. Students who were active in PA were also less often chronically ill than inactive students (17.99 [104/576] vs 23.0% [147/641]; *P*=.04) and an analogous trend was observed among staff members (26.7 [39/145] vs 31.3% [106/340]; *P*=.36). Group differences regarding active or inactive participants in BMI class were identified only for staff members. The physically active staff members had a normal weight more often and were less often obese than inactive employees (*P*=.02; Figure 1, top). Age had no influence on PA in either group of participants.

### Interest in Digital Health Technologies

Data on interest in mobile fitness apps to increase PA were collected only for those participants of the study who were not sufficiently active according to the WHO recommendations. It was found that 53.2% (341/641) of the respective students and 44.2% (150/340) of staff indicated interest in the offers independently of age, gender, or BMI class (Figure 1, bottom). However, the interest of staff members appeared to be dependent on the extent of previous PA. Of those interested in mobile fitness apps, 30.0% (45/150) were previously inactive, whereas the percentage of previously inactive individuals in the group of those who were not interested was only 15.3% (29/190; *P*=.003).

A total of 89.7% (575/641) and 85.6% (291/340), respectively, of previously inactive students and staff expressed willingness to pay at least some of the costs of such a fitness app. In fact, 37.8% (242/641) of these less-active or inactive students and 35.8% (122/340) of staff already use fitness apps.

**Figure 2:**
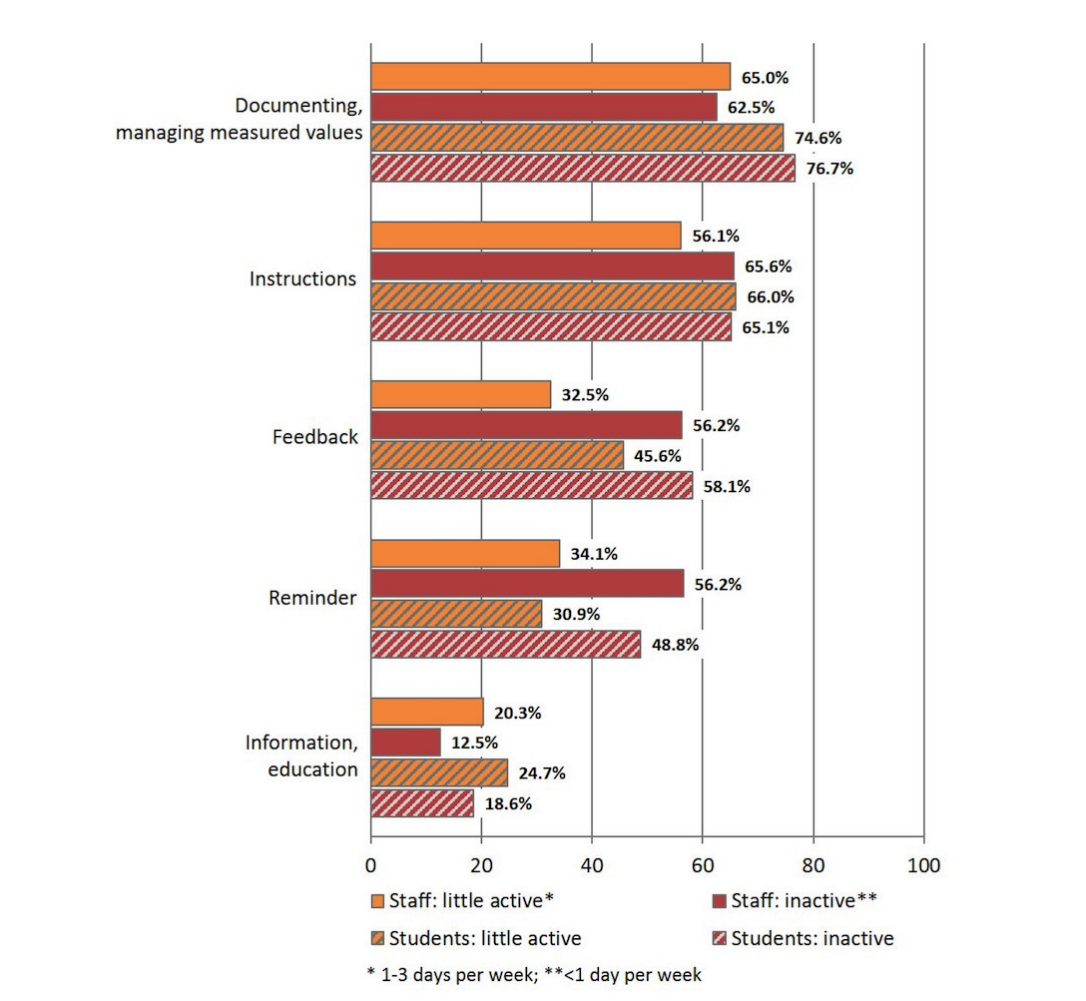
Desired features of mobile fitness apps.

With respect to individual program functions, the greatest interest of those surveyed was in documenting or managing the measured values, instructions, and feedback (Figure 2). With respect to trust in potential providers of health or fitness apps, the majority of the students and staff surveyed were neutral. They were most likely to trust their physician (33%, 212/641, and 27%, 92/340, respectively) and the sports club or trainer (38%, 244/641, and 27%, 92/340, respectively).

Questions as to the potential access to data stored by the health or fitness apps were also answered by most participants with “undecided” or “opposed.” Here as well, the participants would prefer to allow giving access to their physician (53%, 340/641, of students and 45%, 153/340, of staff) or the sports club or trainer (25%, 160/640, and 12%, 41/340, respectively).

## Discussion

### Principal Findings

In this survey, the percentage of physically active university members was high, with 90% of students and over 80% of employees. Of these, 57% of the male and 43% of female students and 37% of male and 27% of female staff members met the level of PA of 150 min (or 3 times) a week as recommend by the WHO. Lampert et al [21] confirmed, based on survey data from the Robert Koch Institute from 2009 for the general population, lower percentages of physically active persons, but simultaneously emphasized the key correlation between PA and social status determined by the level of education and income. With this in mind and taking into account the fact that 10% of the students were majoring in a subject related to sports, the high level of PA among the population in the study is not particularly surprising and is comparable with the results of other internal surveys at universities [22,23].

Around half of the students, who were previously not sufficiently physically active, and one-third of the staff indicated an interest in mHealth apps. Although there was no age dependence within the groups of participants, an age-related dependence of interest in digital solutions may be postulated for the intergroup comparison between students and staff. Recent literature provides extensive evidence that younger individuals in various target groups are more interested in mHealth apps and are more often willing to use such devices [24-26]. There has now been a broad discussion of the problems regarding the acceptance of digital devices among older groups, which focused, for example, on the failure to address the needs of these groups and called for the development of apps specifically for these target and age groups in order to increase user acceptance [27,28].

On the other hand, this study identified only negligible differences between students and staff with respect to potentially interesting functions of mHealth apps. Only “documenting and managing measured values” was named by students (approximately 10%) more often than by staff. Overall, these program functions were among the most important features of a mHealth app for both groups aside from “instructions” and “feedback.” This result can most likely be interpreted in view of the current state of technological development and the demand induced by the providers of mHealth apps. Cultural values, such as the current “quantified self” movement, at the center of which is collecting data on various aspects of one’s own life to improve self-understanding, may also play a role [29-31].

When differentiating between the previously inactive students and staff members compared with those who got PA once to twice a week, in this survey, the “reminder” function that was of considerably greater interest for the inactive individuals, was especially significant. The reminder feature can be considered a self-check technique and supports adherence. It can be assumed that the inactive individuals are well-aware of their need for PA and thus seek suitable support options to adjust their behavior. Similar behavioral patterns are already known from obesity research [32].

Regarding trust in the providers of health or fitness apps and with respect to access to the values measured by such apps, it was found that the great majority of those surveyed were undecided or opposed. They were most likely to trust their own physician and the sports club or trainer in this respect. The indecision and opposition expressed are plausible in view of the requirements of trustworthy apps expressed in literature. Dennison et al [33], for example, emphasize that potential users’ desire confidentiality, expertise of the app providers, and the required transparency regarding the functions of the app and with respect to records of the values measured. Recent studies have shown extensive evidence of general concerns regarding data privacy; here again, some correlation with age and level of education has been postulated in addition to media- and experience-based mistrust [26,34,35].

Willingness to assume the partial or full costs for a mHealth app was generally high among the individuals surveyed who were interested in electronic media, corresponding with a recent study [36]. However, the general willingness expressed in an abstract context does not allow a valid conclusion to be drawn about a concrete decision, which may be more complex and take contextual factors such as the financial burden associated with this purchase into consideration. For example, Dennison et al [33] showed that students expect a health or fitness app to be reasonably priced. Ultimately, the actual willingness to assume the costs of a mHealth app is likely to depend not only on the cost, but also on the anticipated benefit of the concrete app.

### Limitations

The response rate for the study was within the usual range for surveys of this kind. Return rates of less than 10% for students and less than 20% for staff were also reported in other studies of PA at universities [22,23]. Nevertheless, it must be assumed that this level of response rates may be associated with a sampling bias. Furthermore, a sampling bias regarding the gender distribution of the investigated population can be assumed. The male proportion among the respondents was approximately 33% both in students and staff members, whereas the proportion of male is higher in the whole population, that is, 42% of all students and 44% of the entire staff of the university in 2015 were male.

The percentage of physically active students is relatively high in this study at 90% and thus comparable with the results of Preuß et al [22], who found around 80% of physically active students at the University of Bonn. It is assumed that the percentage of physically active students at the University of Potsdam was overestimated due to the high percentage of sport science programs. It is assumed that questions about PA are more likely to be answered by individuals interested in sports. This applies analogously to the group of staff members with 82% of physically active individuals, although comparable data are available in literature here as well [23].

This study has an explorative nature; the results presented should therefore not be simply transferred to all students and staff at the University of Potsdam or to other populations (such as to population-based cohorts).

### Conclusions

Even in younger groups with a high level of education, the majority of individuals do not meet the level of PA recommended by the WHO. However, around half of the individuals in this group of inactive individuals showed interest in mobile apps to encourage activity, indicating that the personalized and age-group-specific use of mHealth apps for optimizing lifestyle through more PA could become increasingly more important.

## Abbreviations

BMI: body mass index
mHealth: mobile health
PA: physical activity
UP: University of Potsdam
WHO: World Health Organization
